# Supplementary material for: Searching Anti-Zika Virus Activity in 1H-1,2,3-Triazole Based Compounds
Source: Molecules. 2021 Sep 28;26(19):5869. doi: 10.3390/molecules26195869 (PMC8510480; doi:10.3390/molecules26195869)
Supplement: Supplementary file 1 [file molecules-26-05869-s001.zip › molecules-1337176-supplementary.pdf]

# Supplementary Materials: Searching Anti-Zika Virus Activity in 1*H*-1,2,3-triazole based compounds

**Willyenne M. Dantas [1,2], Valentina N. M. de Oliveira [3,4], Diogo A. L. Santos [4], Gustavo Seabra [4], Prem Prakash Sharma[5], Brijesh Rathi [5], Lindomar J. Pena [2,*] and Ronaldo N. de Oliveira [1,*]**

[1] Department of Chemistry, Federal Rural University of Pernambuco, Recife 52171-900, Brazil; wil-lyenne.dantas@ufrpe.br (W.M.D.); ronaldo.noliveira@ufrpe.br (R.N.O.)
[2] Department of Virology, Aggeu Magalhães Institute (IAM), Oswaldo Cruz Foundation (Fiocruz), Recife 50670-420, Brazil; lindomar.pena@cpqam.fiocruz.br (L.J.P.)
[3] Instituto Federal de Educação Ciência e Tecnologia de Pernambuco, Campus Ipojuca, 55590-000, Brazil; valentinamelo@ipojuca.ifpe.edu.br (V.N.M.O.);
[4] Department of Fundamental Chemistry, Federal University of Pernambuco, Recife 50740-540, Brazil; diogoalveslopessantos@gmail.com (D.A.L.S.); gustavo.seabra@gmail.com (G.S.)
[5] Laboratory for Translational Chemistry and Drug Discovery, Department of Chemistry, Hansraj College University Enclave, University of Delhi, Delhi 110021, India; premprakash.cr@gmail.com (P.P.S.); brijeshrathi@hrcc.du.ac.in (B.R.)
* Correspondence: ronaldo.noliveira@ufrpe.br (R.N.O.), lindomar.pena@cpqam.fiocruz.br (L.J.P.);
Tel.: +55(81) 3320-6317 (R.N.O.), +55(81) 2123-7849 (L.J.P.)

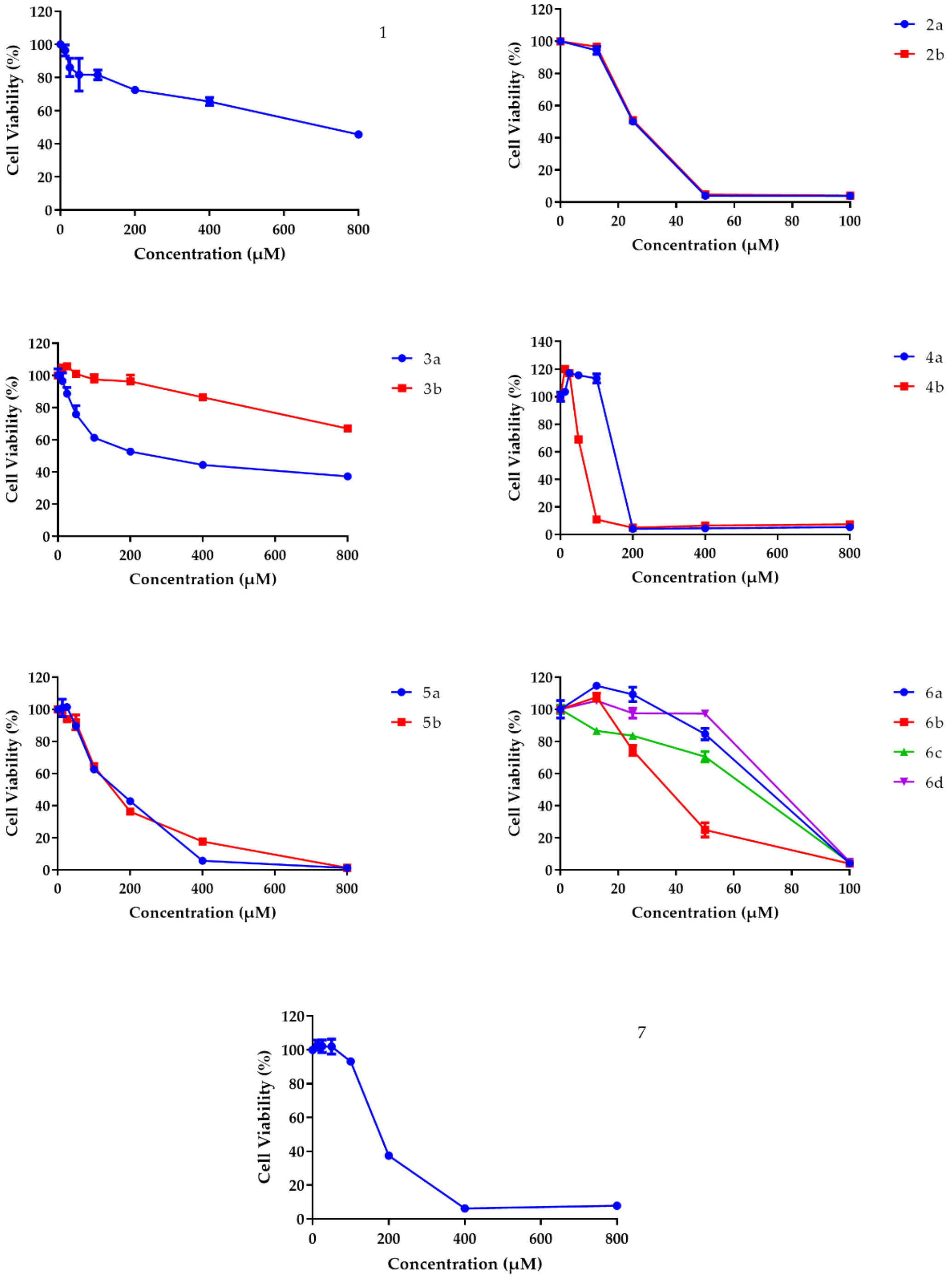


**Figure S1.** Graphical results for cytotoxicity assay of triazole derivates in Vero cells. Cell viability was determined by the MTT method and statistical analysis was performed in GraphPad Prism program. In brief, 1x$10^4$ Vero cells were plated in 96-well microplates for 24h. The compounds were distributed in concentrations between 800-12,5 μM and incubated for 5 days. After reading the ODs by MTT method on spectrophotometer at 570nm, the graphical formation and determination of CC20 and CC50 was made on GraphPad Pris

**Table S1:** Missing residues in RdRp (*5U04*) protein.

| Sequence | Range |
|---|---|
| YHGSYEAPTQGSASS | 306-320 |
| GIAMTDTTPYGQQRVFKEKVDTR | 341-363 |
| ALGAIFEE | 409-416 |
| MMGKREKKQGEFGKAK | 455-470 |
| DDTAGWDT | 534-541 |
| KVVKVLRPAEGGKTVMDIISRQDQRGSGQ | 577-605 |
| RKDTQ | 690-694 |

**Table S2:** Model generated after filling loops for RdRp protein (5U04).

| **Entry** | **Code** | **ERRAT** | **PROSA** | **VERIFY -3D** | **PROCHECK** | | | |
|---|---|---|---|---|---|---|---|---|
| | | | | | **Favoured** | **Additional Favoured** | **Generously Favoured** | **Disallowed** |
| 1 | Model-1 | 64.2361 | -7.42 | 77.42% | 87.8% (462) | 8.9% (47) | 1.9% (10) | 1.3% (7) |
| 2 | Model-2 | 65.2324 | -7.43 | 81.61% | 89.5% (471) | 8.7% (46) | 0.8% (4) | 1.0% (5) |
| 3 | Model-3 | 64.9573 | -7.18 | 82.94% | 88.8% (467) | 9.1% (48) | 1.5% (8) | 0.6% (3) |
| 4 | Model-4 | 70.7612 | -7.82 | 79.77% | 90.9% (478) | 7.0% (37) | 1.0% (5) | 1.1% (6) |
| 5 | Model-5 | 68.4119 | -7.03 | -79.26% | 88.2% (464) | 10.1% (53) | 1.3% (7) | 0.4% (2) |
| 6 | Model-6 | 60.7639 | -5.77 | -72.91% | 87.1% (458) | 8.7% (46) | 2.3% (12) | 1.9% (10) |
| 7 | Model-7 | 62.9948 | -6.98 | 80.43% | 88.0% (463) | 8.9% (47) | 1.7% (9) | 1.3% (7) |
| 8 | Model-8 | 69.4017 | -7.26 | 84.62% | 88.2% (464) | 9.7% (51) | 1.3% (7) | 0.8% (4) |
| 9 | **Model-9** | **70.5882** | **-7.69** | **84.95%** | **89.5% (471)** | **7.8% (41)** | **1.5% (8)** | **1.1% (6)** |
| 10 | Model-10 | 60.0349 | -5.51 | 82.94% | 89.4% (470) | 8.6% (45) | 1.3% (7) | 0.8% (4) |
| 11 | Model-11 | 63.6522 | -7.62 | 78.93% | 87.3% (459) | 9.5% (50) | 1.5% (8) | 1.7% (9) |
| 12 | Model-12 | 63.1488 | -6.46 | 76.09% | 87.8% (462) | 9.7% (51) | 1.3% (7) | 1.1% (6) |
| 13 | Model-13 | 64.2123 | -6.39 | 87.12% | 89.2% (469) | 8.9% (47) | 1.5% (8) | 0.4% (2) |
| 14 | Model-14 | 69.5502 | -6.28 | 80.60% | 89.2% (469) | 8.7% (46) | 1.0% (5) | 1.1% (6) |
| 15 | Model-15 | 68.3566 | -6.01 | 83.44% | 88.8% (467) | 8.4% (44) | 1.9% (10) | 1.0% (5) |

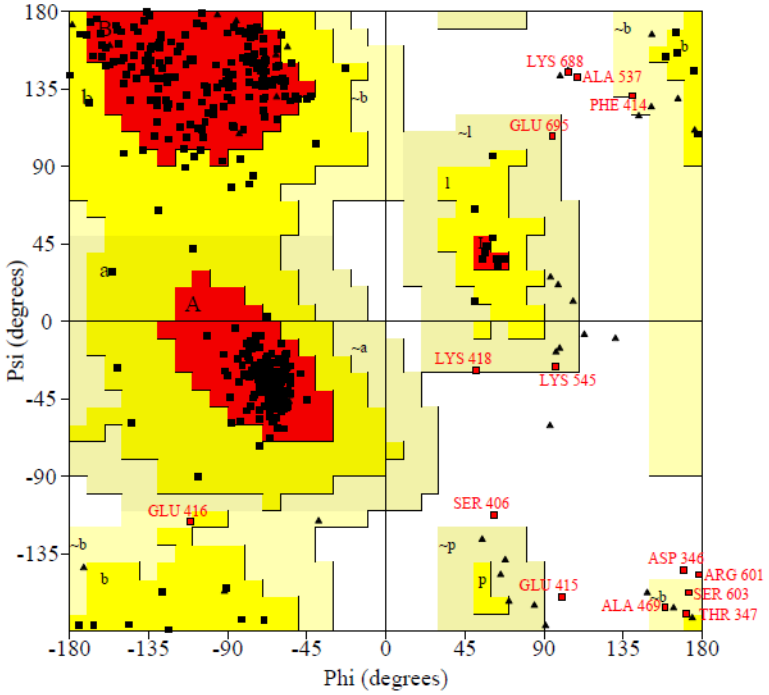


**Figure S2.** Ramachandran map of top scored modeled RdRp (5U04) protein.

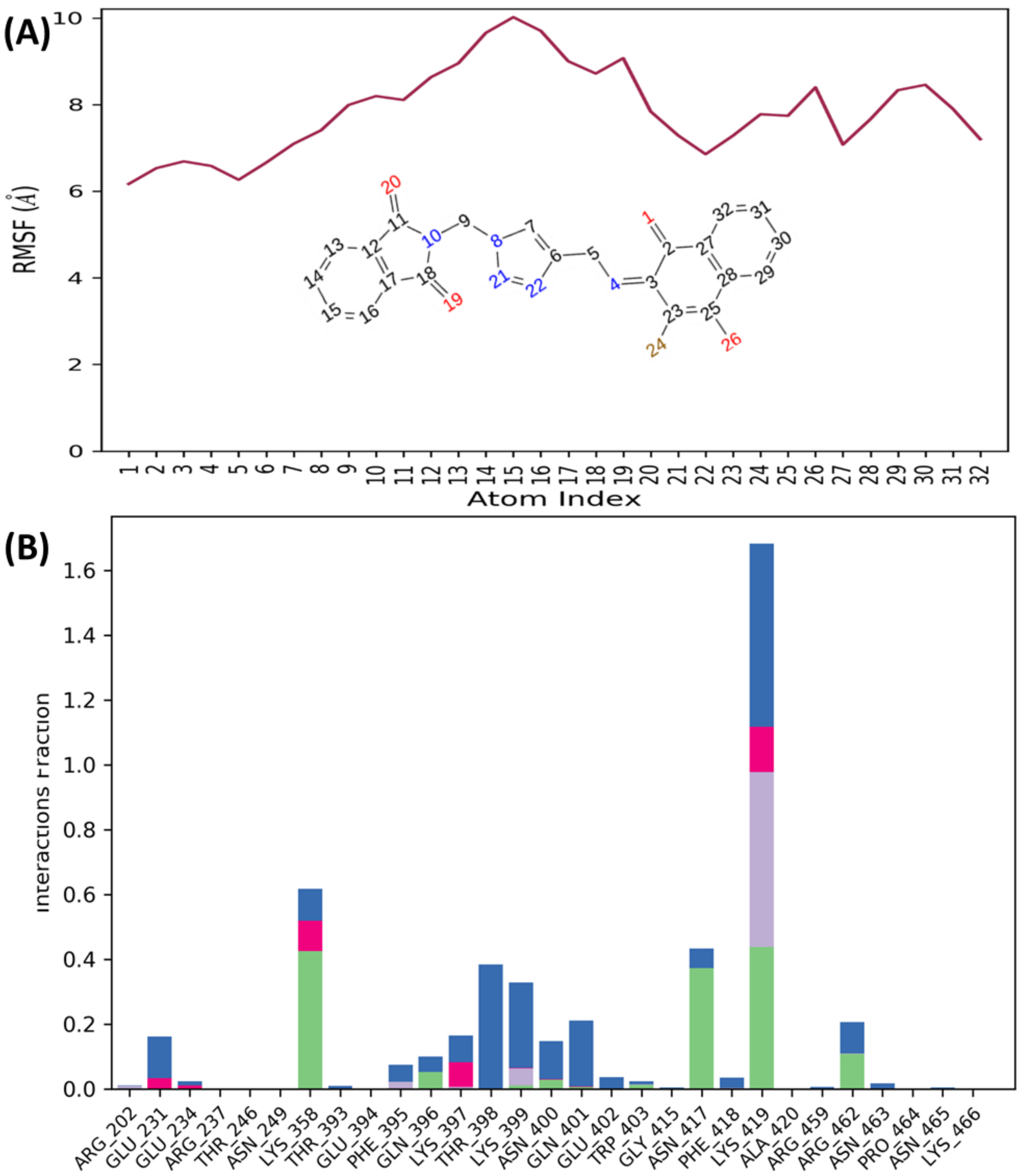


**Figure S3.** MD simulation of **4b**-helicase (6MH3) complex. A) Ligand RMSF-fluctuations of each atom of compound **4b** with respect to protein; B) Histogram plot showed interacting residues with compound **4b** during MD simulation (H-bond: green, hydrophobic: grey, salt bridge interaction: pink, and water bridge interaction: blue).

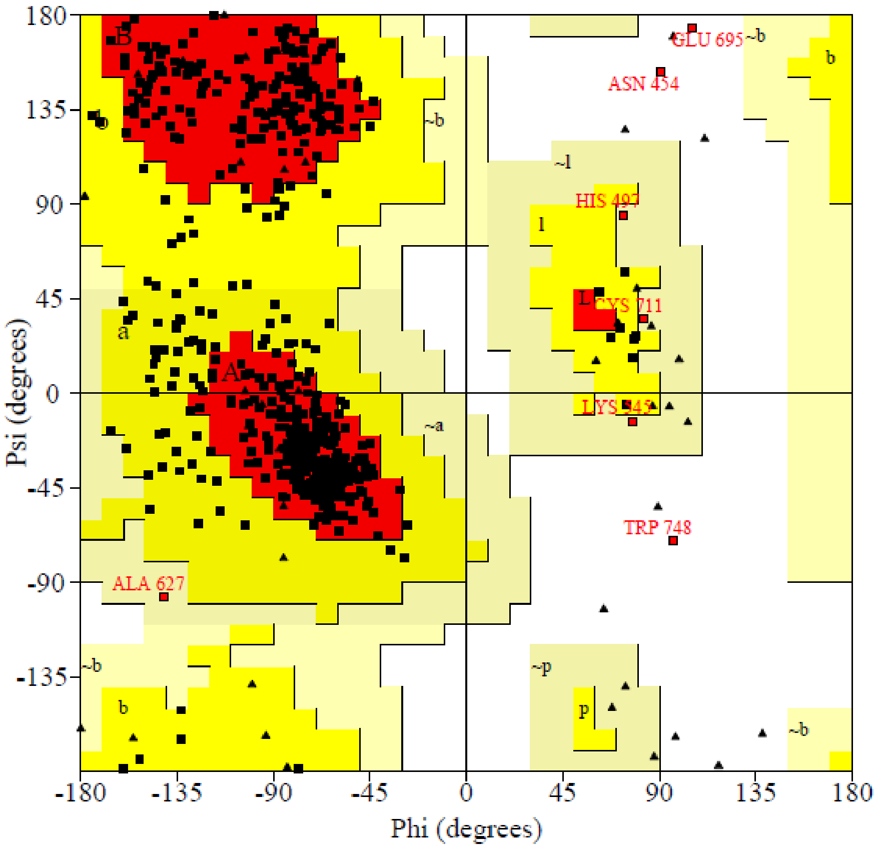


**Figure S4.** Ramachandran map after MD Simulation for **4b**-RdRp (5U04) complex.

**Table S3:** Site score of predicted binding site by Site Map for helicase protein (6MH3).

| Binding Sites | Site Score | Residues |
|---|---|---|
| **Site 3** | **1.097** | **196, 197, 198, 199, 200, 201, 202, 228, 231, 286, 288, 316, 317, 415, 417, 455, 459, 462** |
| Site 1 | 1.055 | 224, 225, 226, 244, 245, 246, 262, 263, 264, 265, 267, 268, 271, 287, 288, 289, 290, 291, 292, 293, 298, 318, 320, 321, 322, 363, 364, 365, 366, 367, 368, 369, 370, 385, 386, 387, 388, 389, 390, 391, 392, 393, 394, 407, 408, 409, 410, 411, 413, 414, 429, 430, 431, 432, 442, 445, 448, 449, 451, 452, 456, 479, 480, 481, 484, 486, 487, 489, 493, 508, 514, 533, 536, 537, 540, 541, 542, 543, 597, 598, 599, 601, 602, 603, 604, 605, 608, 609, 617 |

| | | |
|---|---|---|
| Site 2 | 0.948 | 269, 270, 271, 272, 273, 274, 275, 276, 277, 278, 279, 300, 304, 309, 495, 496, 497, 498, 499, 500, 501, 502, 503, 504, 523, 524, 525, 526, 529, 530, 533, 534, 537, 538 |
| Site 4 | 0.928 | 227, 230, 231, 358, 392, 395, 396, 398, 399, 401, 403, 417, 418, 419, 420, 421, 460, 461, 462, 463, 466, 469 |
| Site 5 | 0.698 | 233, 236, 237, 238, 239, 240, 241, 243, 250, 251, 252, 253, 256 |

**Table S4:** Site score of predicted binding site by Site Map for RdRp protein ((5U04)).

| Binding Sites | Site Score | Residues |
|---|---|---|
| **Site 1** | **1.008** | 338, 339, 340, 341, 342, 343, 344, 345, 346, 416, 417, 418, 420, 486, 490, 513, 533, 534, 535, 536, 539, 540, 541, 542, 543, 544, 545, 547, 548, 551, 570, 573, 574, 575, 576, 604, 605, 607, 608, 609, 611, 612, 620, 623, 663, 664, 665, 666, 667, 676, 691, 702, 709, 710, 711, 712, 713, 714, 731, 732, 734, 735, 738, 739, 742, 743, 744, 745, 746, 747, 748, 749, 752, 753, 756, 760, 763, 768, 792, 793, 794, 795, 796, 797, 798, 799, 800, 801, 803, 805, 806, 848 |
| Site 3 | 1.002 | 358, 359, 360, 361, 362, 363, 365, 368, 633, 636, 637, 681, 684, 685, 689, 692, 693, 695, 696 |
| Site 2 | 0.980 | 758, 762, 765, 769, 772, 775, 776, 779, 782, 785, 786, 787, 788, 808, 809, 810, 811, 812, 834, 835, 836, 840, 858, 883, 884, 885, 886, 887, 888 |
| Site 4 | 0.907 | 400, 403, 404, 412, 413, 415, 416, 417, 419, 422, 454, 455, 459, 465, 470, 471, 472, 473, 476, 479, 483, 487, 491, 494, 495, 602, 603, 604, 605, 606, 607 |
| Site 5 | 0.764 | 354, 355, 356, 358, 626, 627, 629, 631, 632, 633, 675, 678, 679, 680, 681, 696, 698 |

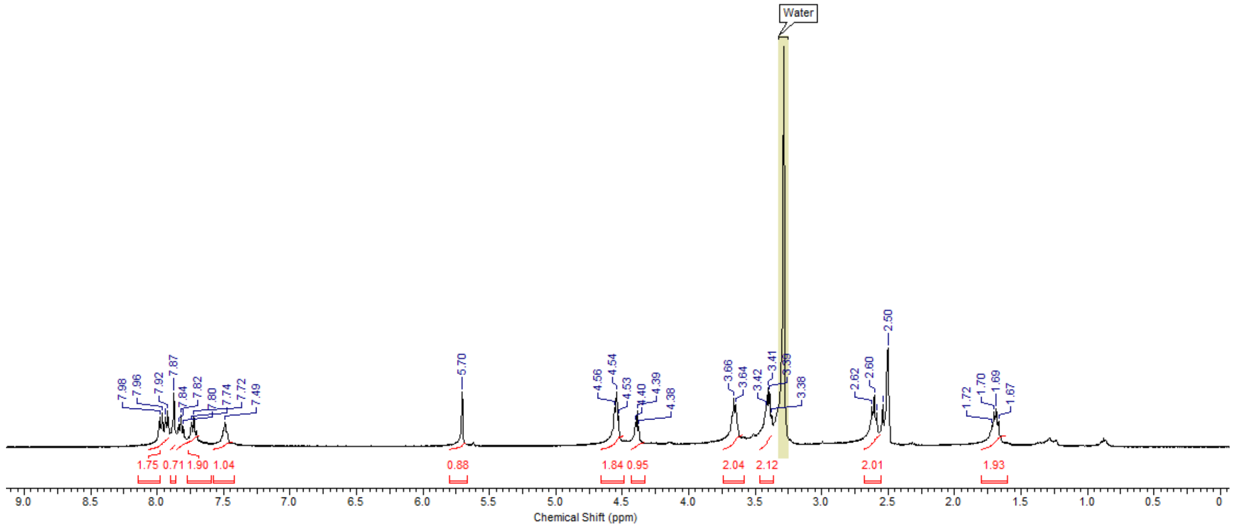


**Figure S5**. $^{1}$H NMR of compound **5a**

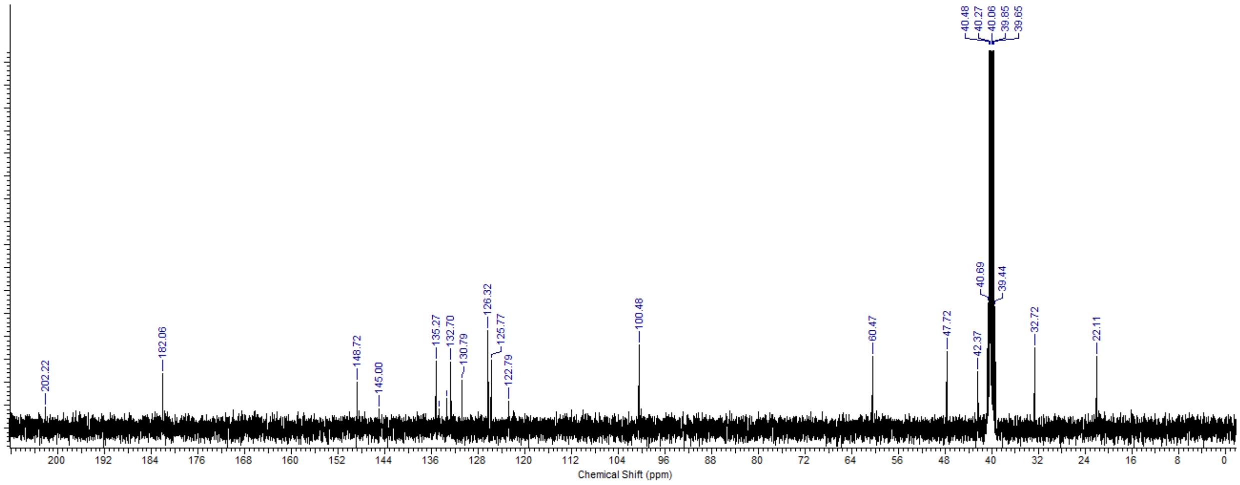


**Figure S6**. $^{13}$C NMR of compound **5a**
